# Coherent Bragg imaging of 60 nm Au nanoparticles under electrochemical control at the NanoMAX beamline

**DOI:** 10.1107/S1600577519010385

**Published:** 2019-08-27

**Authors:** Alexander Björling, Dina Carbone, Francisco J. Sarabia, Susanna Hammarberg, Juan M. Feliu, José Solla-Gullón

**Affiliations:** a MAX IV Laboratory, Lund University, 22100 Lund, Sweden; b Institute of Electrochemistry, University of Alicante, Apdo 99, E-03080 Alicante, Spain; c Synchrotron Radiation Research and NanoLund, Lund University, 22100 Lund, Sweden

**Keywords:** coherent diffraction imaging, nanodiffraction, electrocatalysis

## Abstract

Nanoparticles are essential electrocatalysts in chemical production, water treatment and energy conversion, but engineering efficient and specific catalysts requires understanding complex structure–reactivity relations. Recent experiments have shown that Bragg coherent diffraction imaging might be a powerful tool in this regard. The technique provides three-dimensional lattice strain fields from which surface reactivity maps can be inferred. However, all experiments published so far have investigated particles an order of magnitude larger than those used in practical applications. Studying smaller particles quickly becomes demanding as the diffracted intensity falls. Here, *in situ* nanodiffraction data from 60 nm Au nanoparticles under electrochemical control collected at the hard X-ray nanoprobe beamline of MAX IV, NanoMAX, are presented. Two-dimensional image reconstructions of these particles are produced, and it is estimated that NanoMAX, which is now open for general users, has the requisites for three-dimensional imaging of particles of a size relevant for catalytic applications. This represents the first demonstration of coherent X-ray diffraction experiments performed at a diffraction-limited storage ring, and illustrates the importance of these new sources for experiments where coherence properties become crucial.

## Abstract 2

Coherent imaging in the Bragg geometry of nanoparticles inside an electrochemical cell is presented.

## 1. Introduction   

Nanoparticles are used as catalysts for a range of electrochemical reactions (Kleijn *et al.*, 2014 ▸). For example, the hydrogen fuel cell relies on small dispersions of noble metals to catalyze charge-transfer at both anode and cathode (Katsounaros *et al.*, 2014 ▸). The activity and selectivity of such catalysts depend on particle shape and size, as surface-structural diversity and electronic size effects become more pronounced for smaller particles (Koper, 2011 ▸). For well defined model particles, these reactivity patterns can be compared with single-crystal studies and understood in terms of the exposed crystal facets (Vidal-Iglesias *et al.*, 2012 ▸). However, the direct measurement of local catalytic activity across particle surfaces would allow a more detailed understanding of a more general class of electrocatalysts.

Bragg coherent diffraction imaging (BCDI) (Robinson *et al.*, 2001 ▸) has emerged as a promising tool for operando investigation of crystalline nanoparticles in various contexts (Ulvestad *et al.*, 2014 ▸, 2015 ▸; Ulvestad & Yau, 2017 ▸; You *et al.*, 2017 ▸; Li *et al.*, 2019 ▸). Beside retrieving the shape and crystal structure, this method provides information on the strain through the lattice displacement field, empirically accessible via the phase of the reconstructed three-dimensional object (Robinson & Harder, 2009 ▸; Favre-Nicolin *et al.*, 2010 ▸). This information can, in turn, be used to map catalyst particle reactivity and to localize active sites, provided that the reaction induces some local strain in the lattice. Such strain might originate in adsorption, changes in surface morphology or changes in catalyst composition near the surface. While the resolution achievable in BCDI will not always allow distinguishing between such effects through direct imaging of surface structure, the technique is sensitive enough to localize, for example, specific adsorption of reactants. In this way, the catalytic decomposition of dissolved ascorbic acid on gold surfaces was recently mapped across the surfaces of sub-micrometre particles (Ulvestad *et al.*, 2016 ▸). The method was also used under electrochemical control to study the anodic dissolution of silver particles (Liu *et al.*, 2017 ▸). Although a recent study of heterogeneous methane oxidation used slightly smaller platinum particles of ∼200 nm (Kim *et al.*, 2018 ▸), these pioneering papers all report reactivity maps of particles larger than those found in real applications, for example in fuel cells, which are commonly smaller than 10 nm in size (Koper, 2011 ▸).

The main challenges in investigating smaller particles with this approach are (i) the diffraction signal rapidly falling with particle size, and (ii) the need for stabilizing the nanoparticles over the timescale of the experiment and under the influence of the intense beam. The newly commissioned hard X-ray nanoprobe beamline NanoMAX of the MAX IV Laboratory (Johansson *et al.*, 2013 ▸), the first of the new generation of diffraction-limited storage rings (DLSRs) (Tavares *et al.*, 2014 ▸), provides unprecedented coherent flux densities and therefore has the potential to produce high-resolution coherent diffraction patterns from very small crystals. In this study, we report results from two-dimensional BCDI studies performed on 60 nm gold particles inside an electrochemical cell. We estimate by extrapolation that even smaller particles of sizes relevant for real catalytic applications can be studied at this facility. Stabilizing the nanoparticles under the intense beam in a way that preserves their reactivity will remain an important challenge, a problem which can also be solved by allowing for a certain amount of sample instability in data analysis as recently shown by Calvo-Almazán *et al.* (2019 ▸). The present results represent the first experimental evidence of the new capabilities provided by the high-brilliance coherent beams available at DLSRs.

## 2. Experimental setup and methods   

**Figure 1:**
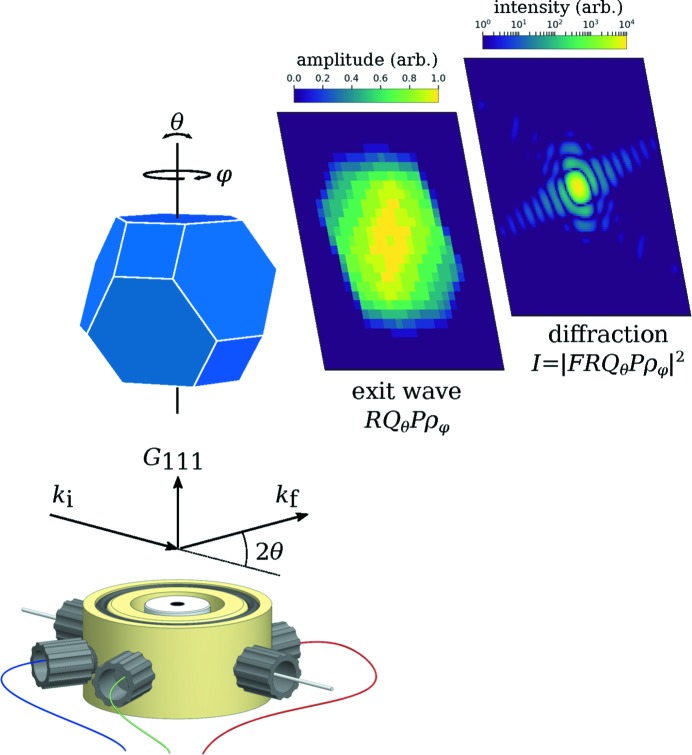
Experimental geometry showing the cell and a truncated octahedron lying on a (111)-type facet, representing a typical orientation of the particles studied. Also shown is the simulated exit wave and diffracted intensity for a given orientation φ assuming flat illumination and perfect alignment with respect to the rocking angle θ. Under these conditions and with the strain-free model particle, the exit wave is real-valued.

Shape-controlled Au nanoparticles were synthesized, cleaned and supported in a conductive carbon matrix as described previously (Kim *et al.*, 2009 ▸; Heo *et al.*, 2008 ▸; Erikson *et al.*, 2014 ▸). The particles were characterized by transmission electron microscopy (TEM) and found to be regular octahedra of side 64 nm, truncated by a cube of side 62 nm (Figure S1 of the supporting information), equivalent in volume to a sphere of diameter 59 nm. A film of nanoparticles in a carbon matrix was deposited on the working electrode of an X-ray compatible electrochemical cell (Figs. S2 and S3), so that the particles were isolated and randomly oriented. The cell was filled with 0.5 *M* H_2_SO_4_, mounted at the NanoMAX diffraction end-station, and the nanoparticles were illuminated by a focused X-ray beam of approximately 100 nm × 100 nm (Fig. S4) produced with a set of KB mirrors. Data acquisition was performed under potentiostatic control at 0.0 V with respect to the Ag/AgCl reference electrode after characterizing the sample by voltammetry (Fig. S5). The scheme of the experimental setup is illustrated in Fig. 1 ▸, which also includes a representation of a model nanoparticle. Coherent diffraction patterns were acquired at the Au(111) Bragg reflection and analyzed using the GPU-accelerated PyNX library (Favre-Nicolin *et al.*, 2019 ▸). Full details on sample preparation, experimental set-up, the electrochemical cell, beam wavefront characterization and the phase retrieval procedure are given in the supporting information.

Following the notation of Hill *et al.* (2018 ▸), the diffracted wavefront is described by the complex exit wave *RQ*_θ_ *P*ρ_φ_. Here, ρ_φ_ is the complex object function at the orientation φ, *P* is the three-dimensional illumination, *Q*_θ_ is a linear phase ramp which encodes the rocking angle θ, and *R* is a projection along the diffracted beam *k*_f_. The measured intensity in the far field is proportional to the square modulus of the Fourier transform *F* of the exit wave, 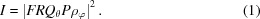
If the rocking angle is zero with respect to the Bragg peak **G**_111_ so that *Q*_θ_ = 1, and if the illumination can be assumed constant across the particle so that *P* = 1, which we can assume for the used beam/particle size ratio, then the exit wave can be approximately considered a projected image *R*ρ_φ_ of the nanoparticle along the exit wave direction *k*_f_.

## 3. Results   

Particles oriented with (111)-type facets in the geometry of Fig. 1 ▸ were found by continuous scanning of the cell through the X-ray beam. Individual particles could not be studied for more than a few seconds since they were found to rotate under the intense beam on that timescale, as illustrated in detail in Fig. S6 of the supporting information. This prevented the acquisition of rocking-curve data from single particles as required to retrieve full three-dimensional information. Particle rotation is a known problem in the study of single nanoparticles with intense beams, caused either by heating or by momentum transfer from beam to particle (Kim *et al.*, 2016 ▸; Liang *et al.*, 2018 ▸). A typical solution, when the particle size and shape is not an issue, is to grow particles on substrates by dewetting (Clark *et al.*, 2013 ▸). Moderate angular instability can be treated with the method recently developed by Calvo-Almazán *et al.* (2019 ▸), which relaxes the requirement for regular spacing along the rocking curve, and which also retrieves the orientations when drift, radiation effects or mechanical imperfections in the instrument cause angular uncertainties. The compromise strategy to collect useful data while minimizing sample instability was to acquire two-dimensional diffraction data from many particles, by attenuating the X-ray beam intensity to ∼10^9^ coherent photons per second and using longer, *i.e.* 3 s, exposure times across a mesh of static positions 3 µm × 3 µm wide. The sample was kept under constant potential as descibed above, and the potential dependence was not further explored. This change of strategy highlights that sample instability is currently a bottleneck for BCDI experiments on smaller nanoparticles at DLSRs, an important challenge to be addressed in future work.

A large number of diffraction spots from different particles were recorded, with the 100 brightest shown in Fig. S7. Some patterns show clear signs of particle rotation during measurement [visible as intensity smearing along the (111) powder ring], whereas others show high-visibility fringes associated with the form factors of static particles. A 3D geometrical model describing the truncated-octahedra nanoparticles (see also Fig. 1 ▸) was created. 2D diffraction data simulated from this model for a range of φ and θ positions (Fig. S8) were found to be in good qualitative agreement with the recorded data.

**Figure 2:**
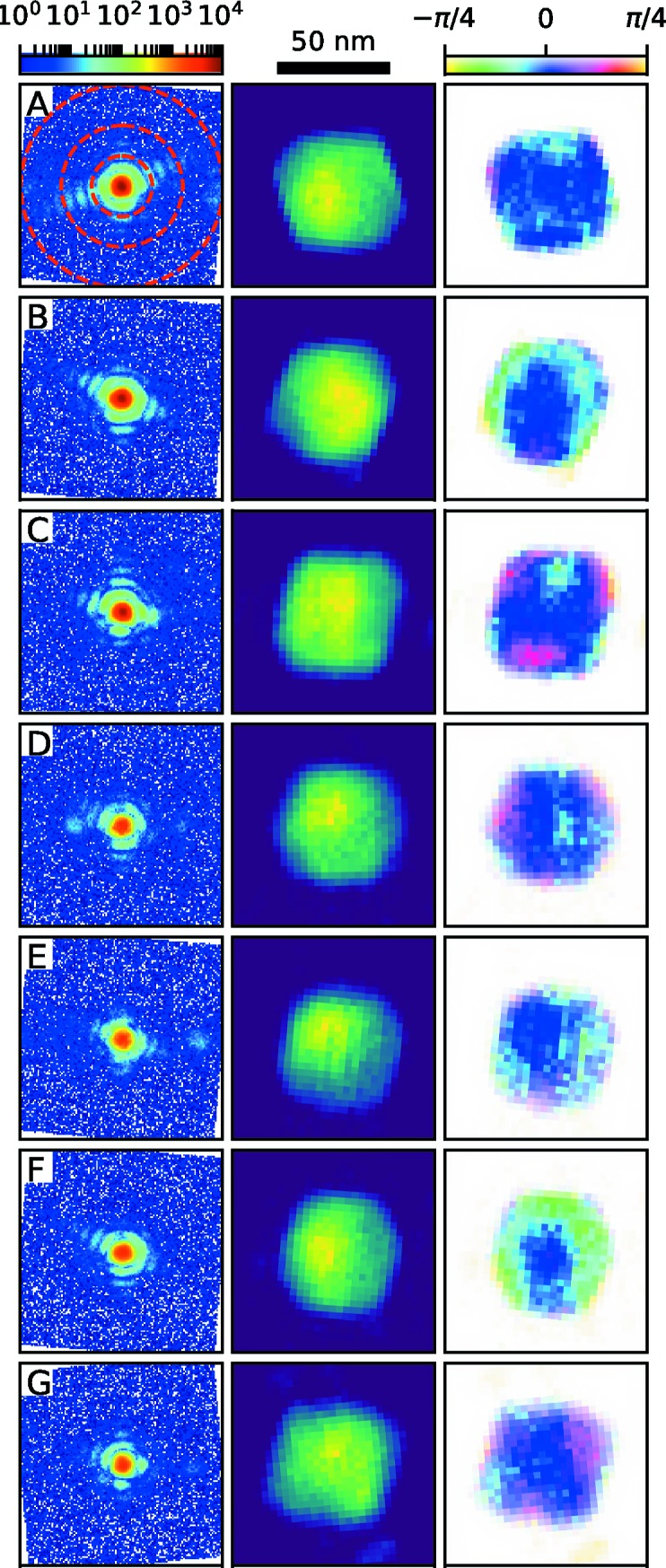
Raw data from seven particle hits (left), together with their reconstructed exit wave amplitudes (middle) and phases (right). Panel A (left) shows single-pixel resolution shells corresponding to 10, 5 and 3 nm. The amplitudes are shown on linear but arbitrary scales and the average phase ramp for each image is removed. Real-space pixel size 3.0 nm.

The 100 brightest particle hits were selected for phase retrieval and assessed as described in the supporting information. It is important to note here that many of the diffraction hits come from particles which rotated during exposure, so that their diffraction patterns do not represent single coherent propagations as described by equation (1). Therefore, high-quality hits were found by selecting the diffraction patterns which gave the most consistent phase retrievals by the chosen figure of merit. The selected hits are shown in Fig. 2 ▸ along with their reconstructions. The reconstructed amplitudes correspond to particles approximately 60 nm in diameter, in agreement with TEM data. Their shapes show clear facets and many (A, B, C and G) are consistent with projected truncated octahedra with φ close to 30° or 90° as seen by comparison with the simulation in Figs. 1 ▸ and S8. Others (D and F) can be recognized as having φ close to 60° (*cf*. Fig. S8). Particles A and C, which are easily related to the TEM-based geometrical model, are found to be described by side 58 nm and 62 nm octahedra truncated by side 62 nm and 63 nm cubes, respectively, in good agreement with the model.

There is considerable structure in the reconstructed phases, with some particles (*e.g.* C, D, G, as well as E and F) showing a threefold symmetric phase pattern. This structure could be related to strain in the particles, caused either by atomic relaxation at edges or by the net negative surface charge at 0.0 V versus Ag/AgCl (Gómez-Marín *et al.*, 2017 ▸). The brightest intensity diffraction hits are expected to come from particles fully illuminated by the focused X-ray beam and with orientations matching the center of the rocking curve, *i.e.* the Bragg condition, so that *Q*_θ_ = 1 and *P* is constant across the particle, the only conditions under which the reconstructions represent projections of the complex object function ρ_φ_ along *k*_f_. However, strain information cannot be reliably extracted from two-dimensional diffraction data. Due to the small beam size, phase structure could come from imperfect beam–particle overlap, which would result in an effective non-uniformity of the beam, *i.e.* variations in *P* across the particle, as simulated in Fig. S9. Also, even small deviations from the Bragg condition, *i.e.* *Q*_θ_ ≠ 1, while not strongly affecting the total intensity, can give rise to phase structure in the reconstructed object (Dzhigaev *et al.*, 2017 ▸), as simulated from the geometrical model in Fig. S8. Therefore, full three-dimensional data are needed to reliably characterize the strain field.

Assuming that the problems of particle instability under the beam can be solved, these results show that operando three-dimensional BCDI of electrocatalytic nanoparticles with size relevant for real application is within reach at the NanoMAX beamline. The instrument currently delivers a coherent flux of 2 × 10^10^ photons s^−1^ focused on a 100 nm × 100 nm spot at 9 keV, or roughly a factor 20 higher than that used in this report. The maximum coherent flux will increase further as the MAX IV storage ring current is increased from 250 mA to the design value of 500 mA (Tavares *et al.*, 2014 ▸; Leemann *et al.*, 2018 ▸) and as the beamline is further optimized, which together will correspond to a factor of 5–10. Even with today’s numbers, diffraction images of the quality reported here can be collected in 180 ms. Assuming that 50 rocking positions are needed for a high-resolution dataset, 3D data could be recorded in 9 s plus overhead due to motor movements. Further, considering that the total diffracted intensity scales with particle size *d* as *d* ^4^, single gold or platinum particles as small as 15 nm could then be imaged in a matter of 45 s in two dimensions or 40 min in three dimensions. This would allow active site localization in, for example, fuel cell catalysis. Future work will focus on addressing particle instability and on exploring potential dependence and dynamics in electrocatalytic systems.

## 4. Related literature   

The following references, not cited in the main body of the paper, have been cited in the supporting information: Bard & Faulkner (2001 ▸); Berenguer *et al.* (2013 ▸); Enders & Thibault (2016 ▸); Fienup (1982 ▸); Gerchberg & Saxton (1972 ▸); Luke (2005 ▸); Mandula *et al.* (2016 ▸); Marchesini *et al.* (2003 ▸); Pérez-Rodriguez *et al.* (2018 ▸); Samant *et al.* (1988 ▸).

## Supplementary Material

Experimental and computational details, supporting figures. DOI: 10.1107/S1600577519010385/gb5090sup1.pdf

## References

[bb31] Bard, A. J. & Faulkner, L. R. (2001). *Electrochemical Methods: Fundamentals and Applications*, 2nd ed. New York: Wiley.

[bb32] Berenguer, F., Godard, P., Allain, M., Belloir, J.-M., Talneau, A., Ravy, S. & Chamard, V. (2013). *Phys. Rev. B*, **88**, 144101.

[bb1] Calvo-Almazán, I., Allain, M., Maddali, S., Chamard, V. & Hruszkewycz, S. O. (2019). *Sci. Rep.* **9**, 6386. 10.1038/s41598-019-42797-4 PMC6477045 31011168

[bb2] Clark, J. N., Beitra, L., Xiong, G., Higginbotham, A., Fritz, D. M., Lemke, H. T., Zhu, D., Chollet, M., Williams, G. J., Messerschmidt, M., Abbey, B., Harder, R. J., Korsunsky, A. M., Wark, J. S. & Robinson, I. K. (2013). *Science*, **341**, 56–59. 10.1126/science.1236034 23704372

[bb3] Dzhigaev, D., Stankevič, T., Bi, Z., Lazarev, S., Rose, M., Shabalin, A., Reinhardt, J., Mikkelsen, A., Samuelson, L., Falkenberg, G., Feidenhans’l, R. & Vartanyants, I. A. (2017). *ACS Nano*, **11**, 6605–6611. 10.1021/acsnano.6b08122 28264155

[bb33] Enders, B. & Thibault, P. (2016). *Proc. R. Soc. London A*, **472**, 20160640. 10.1098/rspa.2016.0640 PMC5247528 28119552

[bb4] Erikson, H., Sarapuu, A., Tammeveski, K., Solla-Gullón, J., Feliu, J. M. & (2014). *ChemElectroChem*, **1**, 1338–1347.

[bb5] Favre-Nicolin, V., Leake, S. & Chushkin, Y. (2019). *arXiv*:1904.07056.

[bb6] Favre-Nicolin, V., Mastropietro, F., Eymery, J., Camacho, D., Niquet, Y. M., Borg, B. M., Messing, M. E., Wernersson, L.-E., Algra, R. E., Bakkers, E. P. A. M., Metzger, T. H., Harder, R. & Robinson, I. K. (2010). *New J. Phys.* **12**, 035013.

[bb34] Fienup, J. R. (1982). *Appl. Opt.* **21**, 2758–2769. 10.1364/AO.21.002758 20396114

[bb35] Gerchberg, R. W. & Saxton, W. O. (1972). *Optik*, **35**, 237–246.

[bb7] Gómez-Marín, A. M., Boronat, A. & Feliu, J. M. (2017). *Russ. J. Electrochem.* **53**, 1029–1041.

[bb8] Heo, J., Kim, D.-S., Kim, Z. H., Lee, Y. W., Kim, D., Kim, M., Kwon, K., Park, H. J., Yun, W. S. & Han, S. W. (2008). *Chem. Commun.* pp. 6120–6122. 10.1039/b815925d 19082092

[bb9] Hill, M. O., Calvo-Almazan, I., Allain, M., Holt, M. V., Ulvestad, A., Treu, J., Koblmüller, G., Huang, C., Huang, X., Yan, H., Nazaretski, E., Chu, Y. S., Stephenson, G. B., Chamard, V., Lauhon, L. J. & Hruszkewycz, S. O. (2018). *Nano Lett.* **18**, 811–819. 10.1021/acs.nanolett.7b04024 29345956

[bb10] Johansson, U., Vogt, U. & Mikkelsen, A. (2013). *Proc. SPIE*, **8851**, 88510L.

[bb11] Katsounaros, I., Cherevko, S., Zeradjanin, A. R. & Mayrhofer, K. J. J. (2014). *Angew. Chem. Int. Ed.* **53**, 102–121. 10.1002/anie.201306588 24339359

[bb12] Kim, D., Chung, M., Carnis, J., Kim, S., Yun, K., Kang, J., Cha, W., Cherukara, M. J., Maxey, E., Harder, R., Sasikumar, K. K. R. S., Sankaranarayanan, S., Zozulya, A., Sprung, M., Riu, D. & Kim, H. (2018). *Nat. Commun.* **9**, 3422. 10.1038/s41467-018-05464-2 PMC6109038 30143615

[bb13] Kim, D., Heo, J., Kim, M., Lee, Y. W. & Han, S. W. (2009). *Chem. Phys. Lett.* **468**, 245–248.

[bb14] Kim, J. W., Ulvestad, A., Manna, S., Harder, R., Fohtung, E., Singer, A., Boucheron, L., Fullerton, E. E. & Shpyrko, O. G. (2016). *J. Appl. Phys.* **120**, 163102.

[bb15] Kleijn, S. E. F., Lai, S. C. S., Koper, M. T. M. & Unwin, P. R. (2014). *Angew. Chem. Int. Ed.* **53**, 3558–3586. 10.1002/anie.201306828 24574053

[bb16] Koper, M. T. M. (2011). *Nanoscale*, **3**, 2054–2073. 10.1039/c0nr00857e 21399781

[bb17] Leemann, S., Sjöström, M. & Andersson, Å. (2018). *Nucl. Instrum. Methods Phys. Res. A*, **883**, 33–47.

[bb18] Li, L., Xie, Y., Maxey, E. & Harder, R. (2019). *J. Synchrotron Rad.* **26**, 220–229. 10.1107/S1600577518016697 30655488

[bb19] Liang, M., Harder, R. & Robinson, I. (2018). *J. Synchrotron Rad.* **25**, 757–762. 10.1107/S1600577518005039 PMC5929357 29714185

[bb20] Liu, Y., Lopes, P. P., Cha, W., Harder, R., Maser, J., Maxey, E., Highland, M. J., Markovic, N. M., Hruszkewycz, S. O., Stephenson, G. B., You, H. & Ulvestad, A. (2017). *Nano Lett.* **17**, 1595–1601. 10.1021/acs.nanolett.6b04760 28186775

[bb36] Luke, D. R. (2005). *Inverse Probl.* **21**, 37–50.

[bb37] Mandula, O., Elzo Aizarna, M., Eymery, J., Burghammer, M. & Favre-Nicolin, V. (2016). *J. Appl. Cryst.* **49**, 1842–1848.

[bb38] Marchesini, S., He, H., Chapman, H. N., Hau-Riege, S. P., Noy, A., Howells, M. R., Weierstall, U. & Spence, J. C. H. (2003). *Phys. Rev. B*, **68**, 140101.

[bb39] Pérez-Rodríguez, S., Pastor, E. & Lázaro, M. J. (2018). *Int. J. Hydrogen Energy*, **43**, 7911–7922.

[bb21] Robinson, I. & Harder, R. (2009). *Nat. Mater.* **8**, 291–298. 10.1038/nmat2400 19308088

[bb22] Robinson, I. K., Vartanyants, I. A., Williams, G. J., Pfeifer, M. A. & Pitney, J. A. (2001). *Phys. Rev. Lett.* **87**, 195505. 10.1103/PhysRevLett.87.195505 11690423

[bb40] Samant, M. G., Toney, M. F., Borges, G. L., Blum, L. & Melroy, O. R. (1988). *J. Phys. Chem.* **92**, 220–225.

[bb23] Tavares, P. F., Leemann, S. C., Sjöström, M. & Andersson, Å. (2014). *J. Synchrotron Rad.* **21**, 862–877. 10.1107/S1600577514011503 PMC4181638 25177978

[bb24] Ulvestad, A., Sasikumar, K., Kim, J. W., Harder, R., Maxey, E., Clark, J. N., Narayanan, B., Deshmukh, S. A., Ferrier, N., Mulvaney, P., Sankaranarayanan, S. K. R. S. & Shpyrko, O. G. (2016). *J. Phys. Chem. Lett.* **7**, 3008–3013. 10.1021/acs.jpclett.6b01038 27429219

[bb25] Ulvestad, A., Singer, A., Cho, H.-M., Clark, J. N., Harder, R., Maser, J., Meng, Y. S. & Shpyrko, O. G. (2014). *Nano Lett.* **14**, 5123–5127. 10.1021/nl501858u 25141157

[bb26] Ulvestad, A., Singer, A., Clark, J. N., Cho, H. M., Kim, J. W., Harder, R., Maser, J., Meng, Y. S. & Shpyrko, O. G. (2015). *Science*, **348**, 1344–1347. 10.1126/science.aaa1313 26089511

[bb27] Ulvestad, A. & Yau, A. (2017). *Nat. Commun.* **8**, 1376. 10.1038/s41467-017-01548-7 PMC5680230 29123126

[bb28] Vidal-Iglesias, F. J., Arán-Ais, R. M., Solla-Gullón, J., Herrero, E. & Feliu, J. M. (2012). *ACS Catal.* **2**, 901–910.

[bb29] You, H., Liu, Y., Ulvestad, A., Pierce, M. S. & Komanicky, V. (2017). *Curr. Opin. Electrochem.* **4**, 89–94.

