# Supplementary material for: Coherent Bragg imaging of 60 nm Au nanoparticles under electrochemical control at the NanoMAX beamline
Source: J Synchrotron Radiat. 2019 Aug 27;26(Pt 5):1830–4. doi: 10.1107/S1600577519010385 (PMC6730624; doi:10.1107/S1600577519010385)
Supplement: Supplementary file 1 [file s-26-01830-sup1.pdf]

Supporting Information:
Coherent Bragg imaging of 60 nm Au nanoparticles under electrochemical control at the NanoMAX beamline

Alexander Björling,[a*] Dina Carbone,[a] Francisco J. Sarabia,[b] Susanna Hammarberg,[c] Juan M. Feliu[b] and José Solla-Gullón[b]

[a]*MAX IV Laboratory, Lund University, 22100 Lund, Sweden,* [b]*Institute of Electrochemistry, University of Alicante, Apdo. 99, E-03080 Alicante, Spain, and* [c]*Synchrotron Radiation Research and NanoLund, Lund University, 22100 Lund, Sweden. E-mail: alexander.bjorling@maxiv.lu.se*

1. Experimental details

1.1. Preparation and cleaning of Au nanoparticles

Carbon supported shaped Au nanoparticles were prepared using a methodology previously described in Kim *et al.* (2009), Heo *et al.* (2008) and Erikson *et al.* (2014). Very briefly, 100 μL of a 100 mM ascorbic acid (Aldrich) aqueous solution was added to a 20 mL aqueous solution containing 1.25×10^{-4} M $HAuCl_4$ and 10 mM cetyltrimethylammonium bromide (CTAB, Aldrich). To this mixture, an aqueous solution of NaOH (100 mM, 100 μL) was quickly injected to induce particle formation. The reaction temperature was maintained at 25 °C and the reaction was allowed to proceed for 20-30 min. Subsequently, the appropriate amount of Vulcan XC-72R carbon was added to the solution. Then, a methanolic solution in which some NaOH pellets were previously dissolved was added to the mixture. The incorporation of this solution induces

the destabilization of the CTAB adsorbed on the surface of the Au nanoparticles, thus producing the precipitation of the sample. Consequently, the sample was filtered, copiously rinsed with ultrapure water and finally dried at 70-80 °C

1.2. TEM characterisation

Transmission electron microscopy (TEM) experiments were performed with a JEOL, JEM-2010 microscope working at 200 kV. The sample for TEM analysis was obtained by placing a droplet of a previously sonicated ethanolic solution containing a small portion of the sample onto a formvar/carbon coated copper grid and allowing the solvent to evaporate in air at room temperature.

1.3. X-ray data collection and electrochemical characterization

All data were collected at the NanoMAX beamline of the MAX IV Laboratory (Johansson *et al.*, 2013). An X-ray compatible PEEK cell (Samant *et al.*, 1988) containing a glassy carbon working electrode (3 mm diameter, Goodfellow), a compact Ag/AgCl reference electrode (eDAQ ET072) and a Pt wire counter electrode (Goodfellow) was used (Figures 1, S2 and S3).

The working electrode was mechanically polished with alumina (0.3 m (Buehler)) and rinsed with ultra-pure water to eliminate impurities and nanoparticles from previous runs. A 4 μL aliquot of aqueous suspension of carbon supported Au nanoparticles was then deposited on the elctrode surface, which was protected and dried under an Ar atmosphere during the evaporation of the solvent. The cell was assembled and filled with 0.5 H_2SO_4 (Merck, Suprapur) before being mounted on the three-axis piezo scanner.

A three-electrode potentiostat (Stanford Research Systems EC301) was integrated into the beamline controls system. Cyclic voltammetry was performed immediately

before X-ray data collection with the sample mounted at the beamline to ensure electrochemical control (Figure S5). All X-ray data were collected at 0.0 V with respect to the reference electrode.

The endstation's Kirkpatrick-Baez mirrors were used to focus the 9.39 keV X-ray beam down to approximately 100 nm by 100 nm FWHM, as found by forward ptychography with a tungsten test structure (Figure S4). Ptychographic reconstruction was carried out using Ptypy (Enders & Thibault, 2016). The cell was rotated by approximately 16° relative to the incoming beam to illuminate the working electrode surface, and a Pilatus 100k detector (Dectris) was held at approximately $2\theta = 32.7°$ to capture Au(111) reflections at a distance of 0.5 m from the sample.

1.4. Phase retrieval

Phase retrieval was carried out using the GPU-accelerated PyNX library (Mandula *et al.*, 2016). For each of the 100 brightest recorded detector frames, 1000 independent reconstructions were run, each with 600 refinement steps of the RAAR algorithm (Luke, 2005) followed by 200 steps of Error Reduction refinement (Gerchberg & Saxton, 1972; Fienup, 1982). The support was updated every 50 steps using the shrink-wrap algorithm (Marchesini *et al.*, 2003). After phase retrieval, the 20 reconstructions with the best free-log-likelihood figures of merit were kept and subjected to mode decomposition analysis (Favre-Nicolin *et al.*, 2019). The first mode, representative of all 20 input images, was considered the reconstructed exit wave for the frame in question. The ratio of total amplitudes between the first and second modes was considered a quality measure of the reconstruction, reflecting the quality of the coherent diffraction pattern itself. Figure 2 shows the frames, among the 100 brightest, with the best ratio between the first and second mode amplitudes.

1.5. Calculation of exit waves and diffraction patterns

All calculations followed Equation 1. The geometrical representation is described in detail in Berenguer *et al.* (2013). All calculations were done in the coordinate system conjugate to the measurement frame, such that the phase ramp Q and the projection R both run parallel to one of the axes, and so that the projection becomes a simple sum. For the calculations in Figure S9, the retrieved probe was extruded along the beam propagation direction and assumed to be constant along the sample (see the depth of focus, Figure S4). For obtaining exit waves, the appopriate phase factor Q_θ was multiplied in before projection, all according to Equation 1. These implementations of the BCDI geometry and of the projection operator are available[1] as a part of the Ptypy package (Enders & Thibault, 2016).

For the geometrical model, the object function was defined for each orientation by first defining the bounding planes of the truncated octahedron, rotating these analytically using the appropriate rotation matrix, and then sampling the octahedron on a discrete grid such that the object function is 1 inside and 0 outside the body. The code which implements these geometrical objects is available online.[2]

The scripts for the specific calculations reported in this paper are available upon request.

[1] http://ptycho.github.io/ptypy/index.html, these implementations are part of the 3dBPP branch, calculations were done at the f972c319 commit.
[2] https://github.com/alexbjorling/nanomax-analysis-utils/blob/master/nmutils/utils/bodies.py

2. Supporting figures

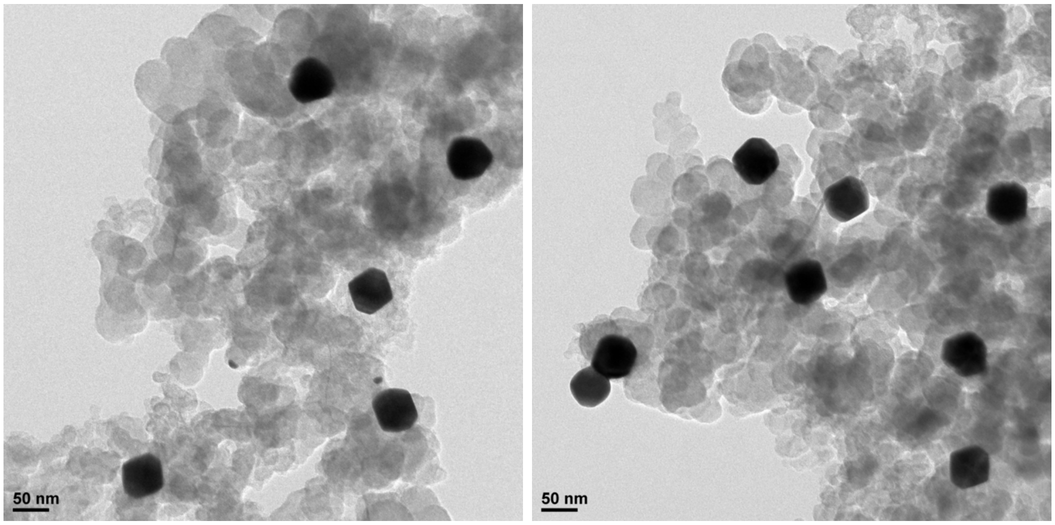

Fig. S1. Transmission electron micrographs of the sample.

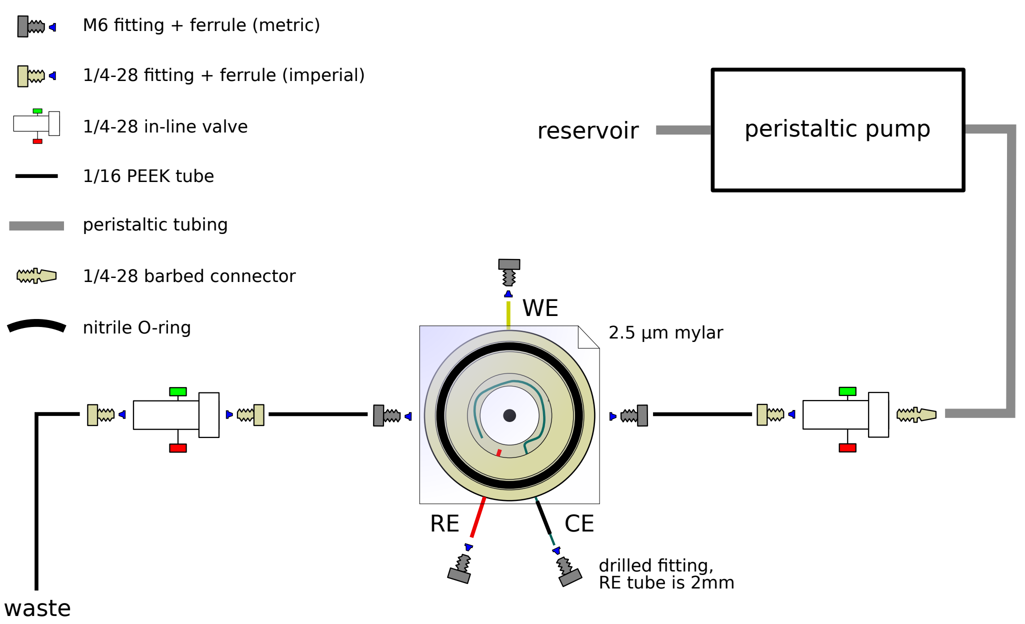

Fig. S2. Schematic drawing of the electrochemical cell assembly. The cell was filled by opening both valves and slowly pumping electrolyte (0.5 M H_2SO_4, Merck) through, making sure to remove any air bubbles. This inflates the mylar window slightly. Then, the outlet valve was closed and the pump reversed, removing the excess liquid from the cell. When the electrolyte layer above the electrode surface was the right thickness, the second valve was closed and the pump stopped. In this way, the electrolyte layer thickness could be adjusted. Its thickness was measured using an optical microscope with a motorized and encoded vertical stage. The thickness varied slightly across the electrode surface because of uneven edges on the teflon holder, but was on the order of 100 μm everywhere. Although a thin layer is preferable from a BCDI point of view, too thin layers can collapse so that the mylar window comes in direct contact with the electrode, in which case conductivity and therefore potential control is lost. The 100 μm approximate thickness was found to be a good compromise. Details of the three electrodes are given above.

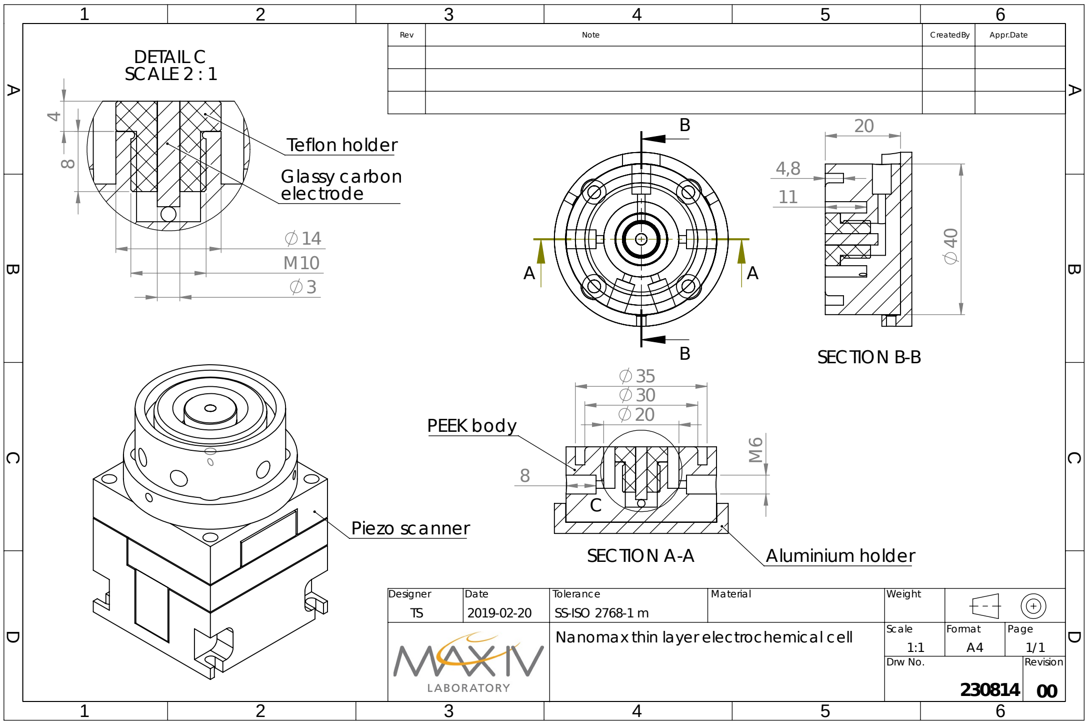

Fig. S3. Technical drawing of the electrochemical cell, including its mounting on the piezo scanner. The cell body is made of PEEK and turned in one piece. The glassy carbon rod is held in a teflon holder, which threads into the PEEK body.

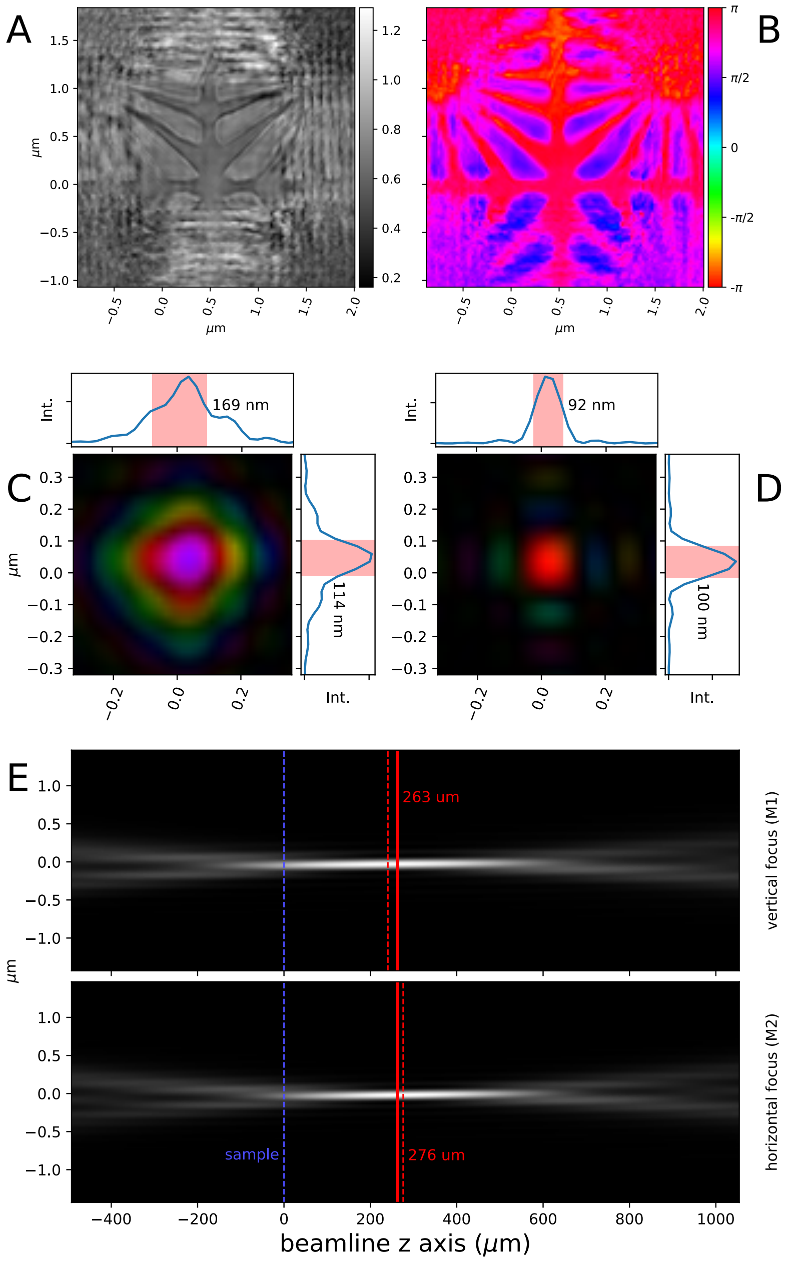

Fig. S4. Ptychographic characterization of the X-ray beam using a 1 μm thick tungsten pattern. A and B respectively show the amplitude and phase of the reconstructed object. C shows the reconstructed probe at the tungsten sample position while D shows the probe numerically propagated to the focal plane where BCDI was later carried out. In E, this propagation is shown in two directions, illustrating the stigmatism of the KB mirrors as well as the depth of focus.

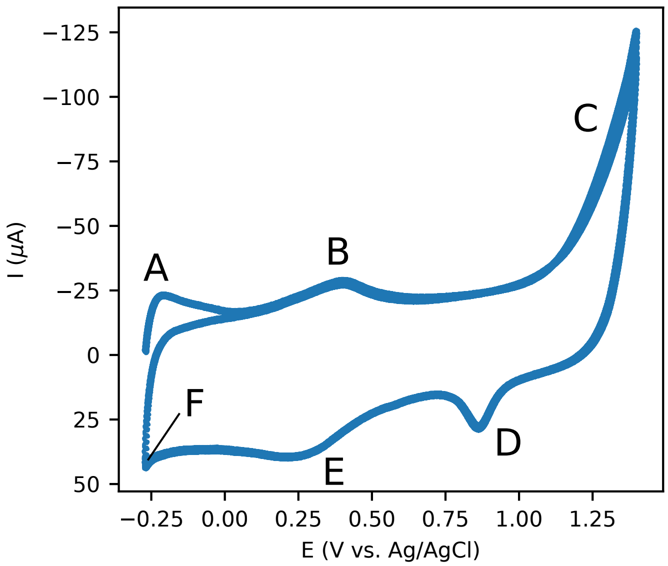

Fig. S5. Cyclic voltammetry (CV) collected before X-ray data acquisition in the X-ray compatible cell (Figure S2). CV is a standard electrochemical characterization technique, where the potential is swept at a steady rate in alternating positive and negative directions, while the current is recorded (Bard & Faulkner, 2001). Scan rate 50 mV/s. The voltammetric curve shows that the Au nanoparticles were under potentiostatic control, and the features are interpreted as follows. A) Oxidation of adsorbed impurities on the first positive excursion, B and E) oxidation and subsequent reduction of oxygen-containing functional groups such as quinones on the Vulcan XC-72R carbon support (Pérez-Rodríguez *et al.*, 2018), C) the beginning of Au surface oxidation, D) subsequent reduction of the partial oxide layer, and F) the very beginning of hydrogen formation. The BCDI experiments were conducted at 0.0 V on this potential scale, in a region where the Au surface is protected from oxidation, the supporting carbon is reduced, and where hydrogen formation still does not occur.

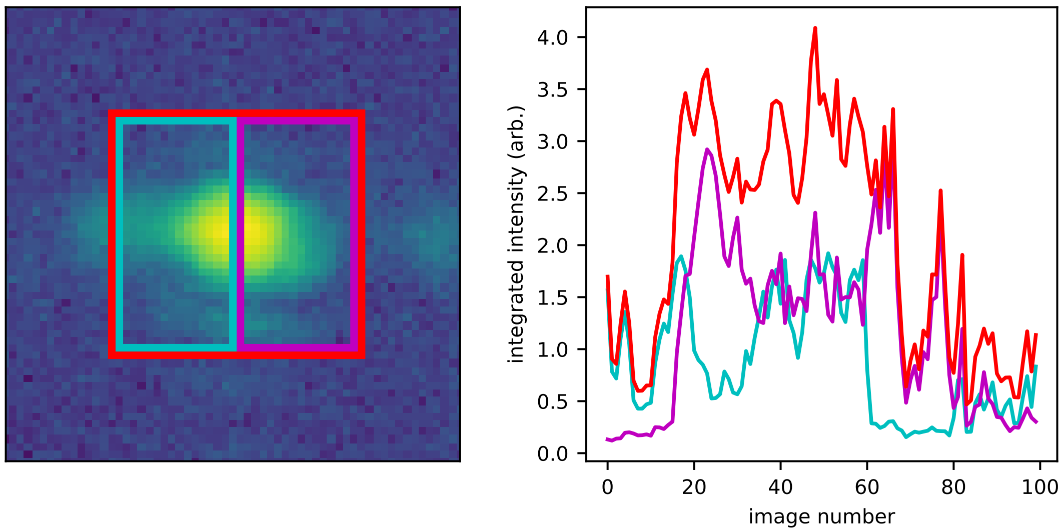

Fig. S6. Sample instability. 100 × 1 s exposures were taken in sequence, illuminating a diffracting particle. Three different regions of the detector image (left) are integrated and plotted (right). Fluctuations in the total intensity (red curve) are associated with rotational instability along θ or with movements laterally through the varying illumination of the X-ray beam. Occasional anti-correlated fluctuations between the turquoise and purple curves correspond to a rotation of the particle along the direction of the powder diffraction ring, such that the diffraction peak moves horizontally in the detector image.

IUCr macros version 2.1.11: 2019/01/14

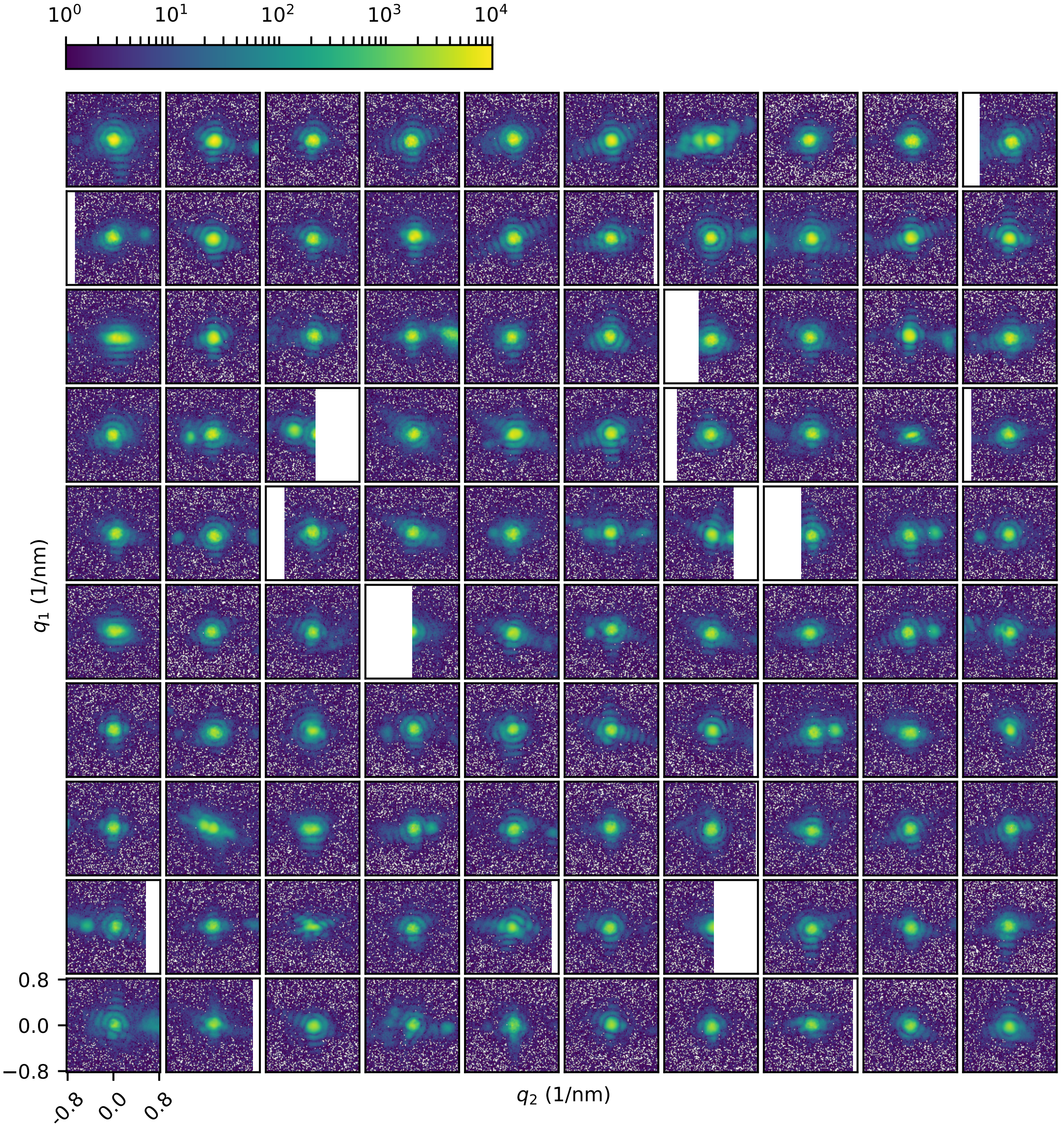

Fig. S7. Raw data. The 100 brightest hits are shown, each frame is cropped to 100 by 100 pixels^2. White fields are areas outside the detector. The definitions of the reciprocal space vectors q_1 and q_2 are found in Berenguer *et al.* (2013), and their scales are given relative to the apparent center of the Bragg peaks.

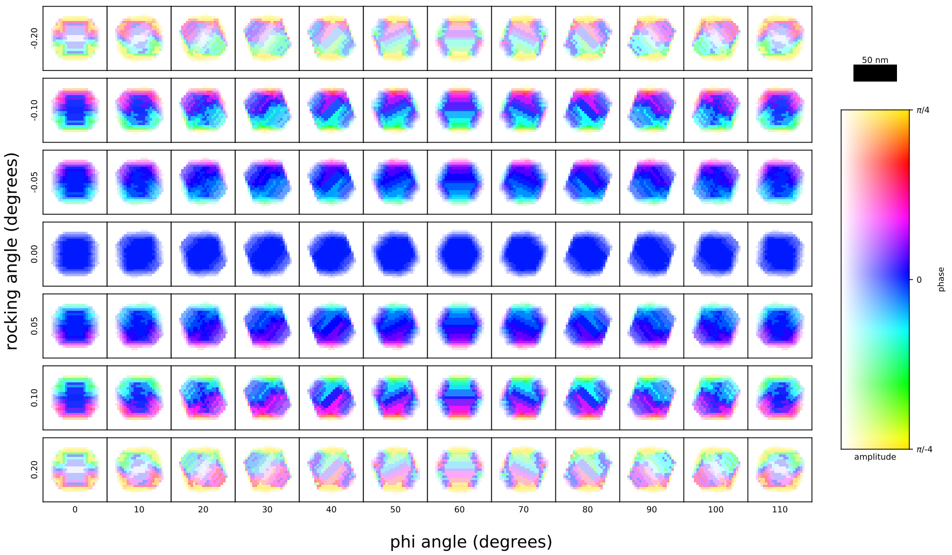

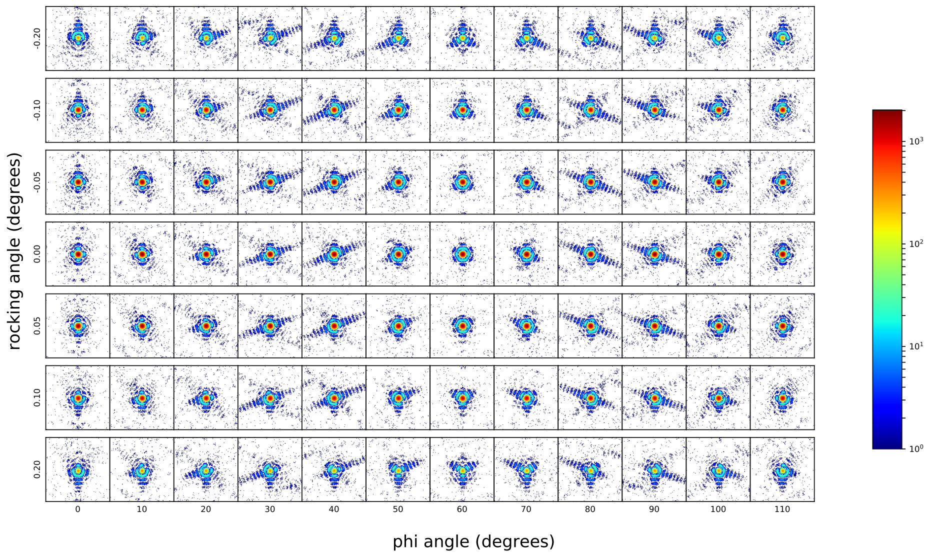

Fig. S8. Exit waves (top) and diffraction patterns (bottom) simulated from the geometrical model based on TEM data, an octahedron with side 63.6 nm truncated by a cube with a side of 62 nm. The simulations assume a flat and constant probe P and reflect a range of orientations and rocking angles as illustrated in Figure 1. Details of the calculations are given above. The diffraction patterns were sampled according to Poisson statistics matching the experimental data.

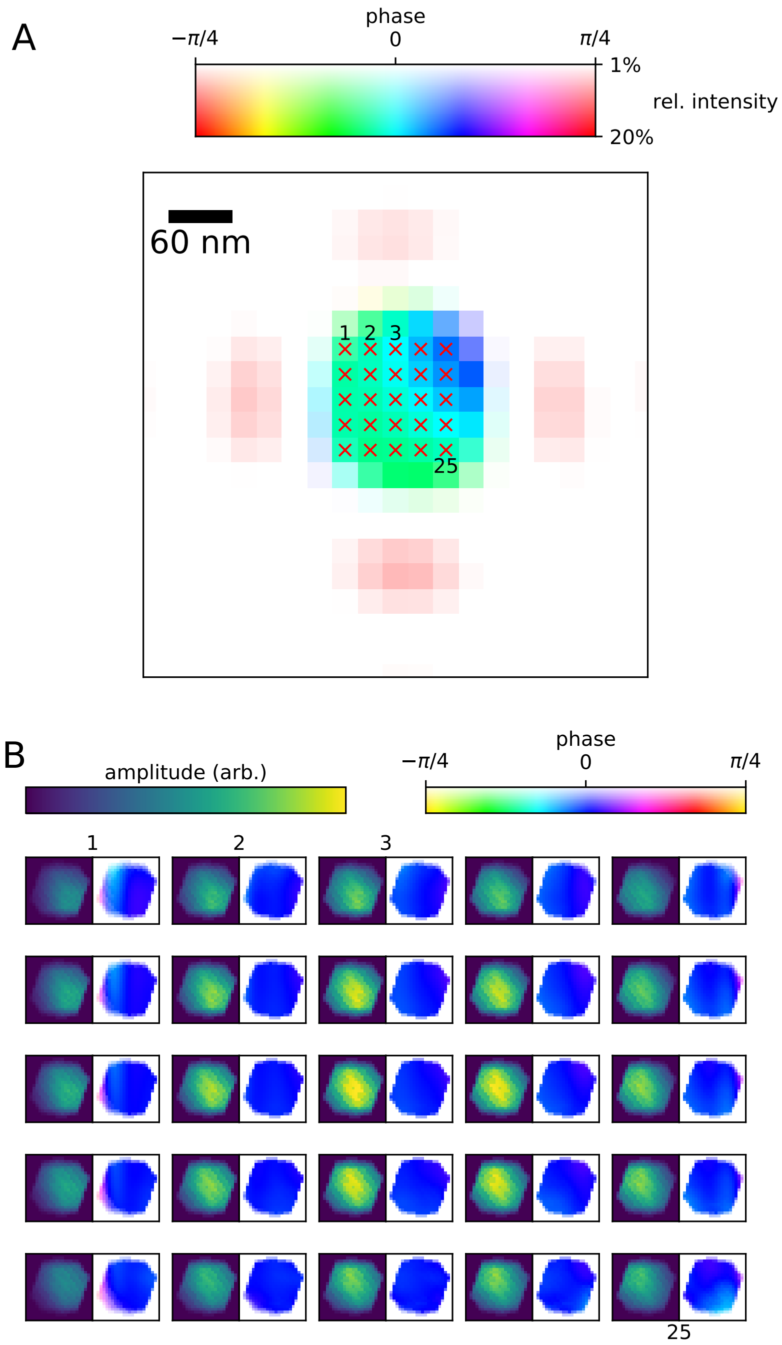

Fig. S9. A. Detailed image of the focused probe as found from ptychography. B. Simulated exit waves from overlapping the retrieved probe with the model particle from Figures 1 and S8 at different positions. The positions are numbered and marked in (A). The strain-free particle corresponds to the orientation for which $\varphi = 30°$ and $Q_\theta = 1$. The exit waves were calculated from the full three-dimensional beam-particle overlap per Equation 1, as described above.

References

Bard, A. J. & Faulkner, L. R. (2001). *Electrochemical methods : fundamentals and applications.* New York: Wiley, 2nd ed.

Berenguer, F., Godard, P., Allain, M., Belloir, J.-M., Talneau, A., Ravy, S. & Chamard, V. (2013). *Phys. Rev. B*, **88**(14), 144101.

Enders, B. & Thibault, P. (2016). *Proc. Royal Soc. Lond. A*, **472**(2196).

Erikson, H., Sarapuu, A., Tammeveski, K., Solla-Gullón, J. & Feliu, J. M. (2014). *ChemElectroChem*, **1**(8), 1338–1347.

Favre-Nicolin, V., Leake, S. & Chushkin, Y. (2019). *Submitted, arXiv:1904.07056 [cond-mat.mtrl-sci].*

Fienup, J. R. (1982). *Appl. Opt.* **21**(15), 2758–2769.

Gerchberg, R. W. & Saxton, W. O. (1972). *Optik*, **35**, 237–246.

Heo, J., Kim, D.-S., Kim, Z. H., Lee, Y. W., Kim, D., Kim, M., Kwon, K., Park, H. J., Yun, W. S. & Han, S. W. (2008). *Chem. Commun.* **0**(46), 6120.

Johansson, U., Vogt, U. & Mikkelsen, A. (2013). *Proc. SPIE*, **8851**, 88510L.

Kim, D., Heo, J., Kim, M., Lee, Y. W. & Han, S. W. (2009). *Chem. Phys. Lett.* **468**(4-6), 245–248.

Luke, D. R. (2005). *Inverse Prob.* **21**(1), 37–50.

Mandula, O., Elzo Aizarna, M., Eymery, J., Burghammer, M. & Favre-Nicolin, V. (2016). *J. Appl. Crystallogr.* **49**(5), 1842–1848.

Marchesini, S., He, H., Chapman, H. N., Hau-Riege, S. P., Noy, A., Howells, M. R., Weierstall, U. & Spence, J. C. H. (2003). *Phys. Rev. B*, **68**(14), 140101.

Pérez-Rodríguez, S., Pastor, E. & Lázaro, M. J. (2018). *Int. J. Hydrog. Energy*, **43**(16), 7911–7922.

Samant, M. G., Toney, M. F., Borges, G. L., Blum, L. & Melroy, O. R. (1988). *J. Phys. Chem*, **92**(1), 220–225.
